# A nomogram based on LI-RADS features, clinical indicators and quantitative contrast-enhanced MRI parameters for predicting glypican-3 expression in hepatocellular carcinoma

**DOI:** 10.3389/fonc.2023.1123141

**Published:** 2023-02-07

**Authors:** Yan Song, Yue-yue Zhang, Qin Yu, Tong Chen, Chao-gang Wei, Rui Zhang, Wei Hu, Xu-jun Qian, Zhi Zhu, Xue-wu Zhang, Jun-kang Shen

**Affiliations:** ^1^ Department of Radiology, The Second Affiliated Hospital of Soochow University, Suzhou, China; ^2^ Department of Radiology, Jieshou City People’s Hospital, Fuyang, China; ^3^ Department of Radiology, Dongtai City People’s Hospital, Yancheng, China; ^4^ Department of Infectious Diseases, Jieshou City People’s Hospital, Fuyang, China; ^5^ Institute of Imaging Medicine, Soochow University, Suzhou, China

**Keywords:** hepatocellular carcinoma, glypican-3, immunotherapy, LI-RADS, Gd-EOB-DTPA, nomogram

## Abstract

**Purpose:**

Noninvasively assessing the tumor biology and microenvironment before treatment is greatly important, and glypican-3 (GPC-3) is a new-generation immunotherapy target for hepatocellular carcinoma (HCC). This study investigated the application value of a nomogram based on LI-RADS features, quantitative contrast-enhanced MRI parameters and clinical indicators in the noninvasive preoperative prediction of GPC-3 expression in HCC.

**Methods and materials:**

We retrospectively reviewed 127 patients with pathologically confirmed solitary HCC who underwent Gd-EOB-DTPA MRI examinations and related laboratory tests. Quantitative contrast-enhanced MRI parameters and clinical indicators were collected by an abdominal radiologist, and LI-RADS features were independently assessed and recorded by three trained intermediate- and senior-level radiologists. The pathological and immunohistochemical results of HCC were determined by two senior pathologists. All patients were divided into a training cohort (88 cases) and validation cohort (39 cases). Univariate analysis and multivariate logistic regression were performed to identify independent predictors of GPC-3 expression in HCC, and a nomogram model was established in the training cohort. The performance of the nomogram was assessed by the area under the receiver operating characteristic curve (AUC) and the calibration curve in the training cohort and validation cohort, respectively.

**Results:**

Blood products in mass, nodule-in-nodule architecture, mosaic architecture, contrast enhancement ratio (CER), transition phase lesion-liver parenchyma signal ratio (TP-LNR), and serum ferritin (Fer) were independent predictors of GPC-3 expression, with odds ratios (ORs) of 5.437, 10.682, 5.477, 11.788, 0.028, and 1.005, respectively. Nomogram based on LI-RADS features (blood products in mass, nodule-in-nodule architecture and mosaic architecture), quantitative contrast-enhanced MRI parameters (CER and TP-LNR) and clinical indicators (Fer) for predicting GPC-3 expression in HCC was established successfully. The nomogram showed good discrimination (AUC of 0.925 in the training cohort and 0.908 in the validation cohort) and favorable calibration. The diagnostic sensitivity and specificity were 76.9% and 92.3% in the training cohort, 76.8% and 93.8% in the validation cohort respectively.

**Conclusion:**

The nomogram constructed from LI-RADS features, quantitative contrast-enhanced MRI parameters and clinical indicators has high application value, can accurately predict GPC-3 expression in HCC and may help noninvasively identify potential patients for GPC-3 immunotherapy.

## 1 Introduction

Hepatocellular carcinoma (HCC) is the most common primary malignancy of the liver. It is the sixth most common cancer and the third leading cause of cancer-related death in the world. There are approximately 910,000 new cases and 630,000 deaths each year, with the disease affecting more males than females, and Asia is considered a high-risk area (1). Several guidelines have noted that the medical imaging findings of HCC are consistent with the typical manifestations of the disease, and the treatment process can be directly selected without a pathological diagnosis (2). Therefore, strengthening the diagnosis and evaluation of HCC before treatment are greatly important. The Liver Imaging Reporting and Data System (LI-RADS) was published by the American College of Radiology. Its main purpose is to standardize image acquisition protocols for liver imaging, interpret features in imaging reports and promote communication between different HCC-related disciplines. LI-RADS has now been fully incorporated into the clinical diagnosis and treatment guidelines of the American Association for the Study of Liver Diseases, reflecting the importance of LI-RADS in the diagnosis and treatment of HCC (3). GPC is a heparan sulfate proteoglycan that is connected to the cell membrane through glycosylphosphatidylinositol anchors. GPC-3 is one of the subtypes and regulates tumor formation, differentiation, and metastasis and the immune microenvironment by participating in multiple signaling pathways (4). Studies have shown that GPC-3 can be used as an immunotherapy target for HCC, and GPC-3-targeting monoclonal antibodies and GPC-3-targeting chimeric antigen receptor T cells (CAR-T) can effectively kill GPC-3-positive cells, including HCC tumor cells (5, 6).

Existing studies have shown that contrast-enhanced MRI-related parameters and features can better predict the expression of Ki-67 (7) and cytokeratin 19 (8) in HCC than other modalities and can predict prognostic microvascular invasion (MVI) (9) and pathological types (10). Other imaging-related feature are also associated with early recurrence of HCC (11). However, studies on the prediction of targets for immunotherapy are insufficient. The accumulation of specific targets or drug carriers in tumors can be visualized by MRI (12, 13), so GPC-3 expression can also be assessed by noninvasive imaging. It has been reported that superparamagnetic iron oxide (SPIO) anti-GPC-3 molecular probes and GPC3-targeted immuno-positron emission tomography (immunoPET) can more accurately evaluate the expression of GPC-3 in HCC tissues (14–16), but SPIO and immunoPET have not yet been widely used. CHEN R et al. (17) demonstrated that preoperative Iterative Decomposition of water and fat with Echo Asymmetry and Least squares estimation (IDEAL IQ) can noninvasively predict GPC-3. Some researchers have also used preoperative MRI-based radiomics to predict the expression of GPC-3 (18, 19). Although they have achieved good results, the above methods require commercial software to transform the results, thus the clinical promotion is limited. Chen et al. (20) enrolled 278 patients with solitary HCC, and analyzed the relationship between EOB-MR imaging features and GPC-3 positive expression, the results showed that serum alpha-fetoprotein >10 ng/ml and five EOB-MR imaging features, including tumor size >3.0cm, non-peripheral “washout”, infiltrative appearance, marked diffusion restriction, and iron sparing in solid mass were significantly associated with positive GPC-3 expression. However, the relationship between quantitative contrast-enhanced MRI parameters and GPC-3 positive expression still needs further study. Although GPC-3 can be released from the surface of HCC cells into the blood, previous studies have shown that the positive rate of GPC-3 in the serum of HCC patients is low, and there is no significant difference in serum GPC-3 levels between patients with small HCC and non-HCC patients (21, 22). Therefore, the diagnostic performance of this laboratory parameter is limited.

At present, the evaluation of GPC-3 expression still relies on invasive postoperative pathological examination, and the results may be affected by the sampling site. Therefore, the aim of this study was to establish and validate a noninvasive, straightforward, and reproducible model to predict GPC-3 expression using LI-RADS features, quantitative contrast-enhanced MRI parameters, and clinical indicators.

## 2 Materials and methods

### 2.1 Study population

**Figure 1:**
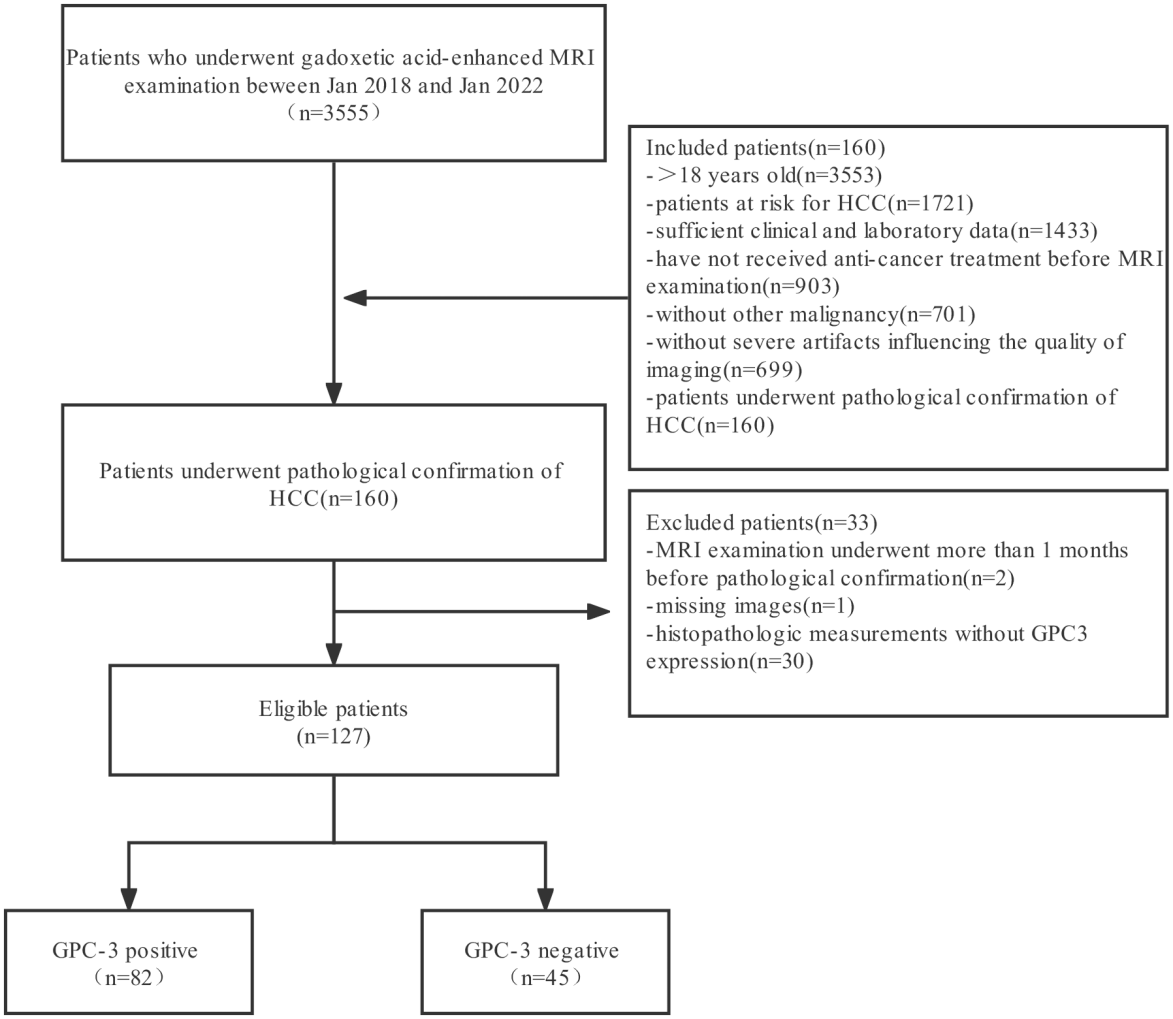
The workflow of patient selection for this study.

Patients who underwent dynamic contrast-enhanced MRI of the abdomen due to risk factors for HCC in the Second Affiliated Hospital of Soochow University from January 2018 to January 2022 were retrospectively collected. The inclusion criteria were as follows: ① patients aged ≥ 18 years; ② patients with chronic HBV hepatitis; ③ patients with liver cirrhosis; and ④ liver transplant donor candidates and recipient candidates. The exclusion criteria were as follows: ① received any form of antitumor therapy or needle biopsy before dynamic contrast-enhanced MRI; ②secondary cirrhosis due to congenital liver fibrosis; ③ cirrhosis due to vascular disease; ④ unclear GPC-3 expression status in postoperative pathological results; and ⑤ poor image quality that affected interpretation or scanning protocol that did not meet the LI-RADS requirements. The patient screening flowchart is shown in **Figure 1**.

### 2.2 Image acquisition

Scanning was performed using a 3.0-T MR scanner (Ingenia, Philips Healthcare, Best, Netherlands); a 32-channel phased-array body coil was used as the receiving coil. The unenhanced sequence included the respiration-triggered T2WI spectral attenuated inversion recovery (SPAIR) sequence along the horizontal axis, the coronal fat suppression sequence and the in-/out-of-phase T1WI data collected by DIXON. The breath-triggered diffusion-weighted imaging (DWI) included multiple b values of 0, 50, 800, 1000, and 1500. Enhanced images were acquired using a modified Dixon (mDixon) sequence, including seven dynamic enhancement phases: pre-enhanced phase (PP), early arterial phase (EAP), standard arterial phase (SAP), late artery phase (LAP), portal venous phase (PVP), transition phase (TP) and hepatobiliary phase (HBP). The slice thickness of the abovementioned sequence was 5 mm, the slice interval was 2-5 mm, the matrix was ​​ (224–512) X (224–512), the field of view was 400, and the inversion angle was 10°/90°. The contrast agent was disodium gadoxetate (Gd-EOB-DTPA; Primovist; Bayer Schering Pharma AG, Berlin, Germany). The contrast agent was intravenously administered *via* a power injector followed by a 25.0-mL saline flush. The contrast agent dosage was 0.1 ml/kg, and the flow rate was 1.0 ml/s. All acquisition technologies complied with LI-RADS v2018 technical requirements.

### 2.3 Clinical data

Clinical data, including age, sex, laboratory findings, and Child−Pugh score, were collected and recorded in an electronic medical record system by radiologists with 9 years of experience in abdominal MRI. The laboratory tests included total bilirubin (TB), direct bilirubin (DBil), aspartate aminotransferase (AST), alanine aminotransferase (ALT), total protein (TP), globulin protein (GLB), prothrombin time (PT), platelet count (PLT), international normalized ratio (INR), alpha-fetoprotein (AFP), carbohydrate antigen 242 (CA242), cytokeratin 19 fragment (CYFRA211), tumor-specific growth factor (TSGF), and Fer.

### 2.4 Image analysis

A radiologist with 9 years of experience in abdominal MRI performed lesion matching and labeling. The label matching method was used to determine the location of the lesion according to the treatment monitoring images, pathological results or follow-up images and identify the mass based on its size, serial number, and image number. The lesion size was measured three times on the HBP, TP/DP, PVP, and T2WI scans according to the measurement method required in LI-RADS, and the average value was taken and recorded in the stored electronic file. The signal intensity (SI) of the lesions, adjacent normal liver tissue, and erector spinae muscle at the same plane were measured in the PP, EAP, SAP, LAP, PVP, TP and HBP. A region-of-interest (ROI, size: 100 mm^2^) was manually set, avoiding visible vessels, bile ducts, hemorrhage, cystic degeneration and imaging artifacts. Each ROI was measured three times, and the mean value was recorded. The formulas used to calculate LNR and CER were as follows: LNR=SI lesion/SI liver, LMR=SI lesion/SI erector spinae muscle and CER=(LNR on LAP–LNR on PP)/LNR on PP.

Three intermediate- and senior-level radiologists (7-11 years of experience in MRI diagnosis) received systematic training of LI-RADS v2018. All 3 radiologists had applied LI-RADS v2018 clinically for at least three months and passed the ACR LI-RADS case assessment. The LI-RADS features of all included lesions were assessed within the same month by three radiologists. The assessment features included nonrim arterial phase hyperenhancement (APHE), non-peripheral washout, enhancing capsule, threshold growth, mosaic architecture, non-enhancing capsule, blood products in mass, fat in mass more than adjacent liver, nodule-in-nodule architecture, corona enhancement, restricted diffusion, mild-to-moderate T2 hyperintensity, fat sparing in solid mass, iron sparing in solid mass, transitional phase hypointensity, hepatobiliary phase hypointensity, and subthreshold growth. All the radiologists were blinded to the clinical data and GPC-3 expression status. During the evaluation process, the radiologists were blinded by each other’s results. Any disputed results were resolved through negotiation at the final intragroup meeting. If the disagreement could not be resolved, the final result was decided by a majority consensus.

### 2.5 Statistical analysis

Statistical analysis was performed by using version 26.0 SPSS (IBM, Armonk, New York, USA). The kappa test was used to analyze the consistency of features recognized between two radiologists. Kendall’s W test was used to compare the consistency of features recognized among the three radiologists. The kappa values and Kendall’s W values were interpreted as follows: poor, 0.00–0.20; fair, 0.21–0.40; moderate, 0.41–0.60; good, 0.61–0.80; and excellent, 0.81–1.00. The continuous variables were compared by the Shapiro−Wilk test; the data that conformed to a normal distribution are expressed as the mean ± standard deviation and were analyzed using two independent samples t test, and those that conformed to a skewed distribution are expressed as the median ± interquartile range and were analyzed using the Mann−Whitney U test. The χ^2^ test was used to compare categorical data between different GPC-3 expression in training cohort. The above factors with p < 0.1 were included in multivariate logistic regression, and the stepwise backward method was adopted to identify independent predictors of positive GPC-3 expression. The nomogram model and calibration curve were constructed using the “rms” package in R software (version 3.6.2, http://www.r-project.org/). The predictive performance of the nomogram model was evaluated by calibration curves and receiver operating characteristic (ROC) curves. P<0.05 indicated statistically significant differences.

## 3 Results

### 3.1 Patients

A total of 127 patients were included in this study, including 95 males (74.80%) and 32 females (25.20%). The age ranged from 20 to 83 years, with a mean of (59.64 ± 13.27) years, and the lesion size ranged from 0.6 cm to 18.9 cm, with a mean of (4.84 ± 3.33) cm. Immunohistochemistry showed positive GPC-3 expression in 82 cases (64.57%) and negative expression in 45 cases (35.43%). There was no significant difference in age, lesion size (continuous variable) or sex ratio between GPC-3 expression in the training cohort and validation cohort (P>0.05). Among the 127 patients, 100 patients (78.7%) had liver cirrhosis. The underlying liver-related causes were HBV infection in 109 patients (85.8%), alcoholic cirrhosis in 5 patients (3.9%), HCV infection in 4 patients (3.1%), and schistosomal cirrhosis in 3 patients (2.4%). Nonalcoholic fatty liver disease was present in 1 patient (0.8%), and 5 patients (3.9%) had liver cirrhosis due to other unknown causes. There were no significant differences in the underlying etiology between GPC-3 expression in the training cohort and validation cohort (P>0.05) (**Table 1**).

**Table 1:** Clinical and pathological characteristics of study patients.

Variables		All cases	training cohort				validation cohort			
			GPC-3 negative	GPC-3 positive	Test values	P values	GPC-3 negative	GPC-3 positive	Test values	P values
Number of patients		127	32	56	–	–	13	26	–	–
Age (years)		59.64 ± 13.27	58.59 ± 13.17	60.38 ± 13.21	t=0.609	0.544	63.08 ± 13.09	57.62 ± 13.88	1.180	0.246
Tumor size (cm)		4.84 ± 3.33	3.91 ± 3.72	5.31 ± 3.06	t=-1.908	0.060	4.57 ± 2.92	5.09 ± 3.52	t=-0.464	0.645
Sex	Male	95 (74.8%)	27 (84.4%)	39 (69.6.2%)	χ^2^ = 2.357	0.125	8 (61.5%)	21 (80.8)	χ^2^ = 1.681	0.253
	Female	32 (25.2%)	5 (15.6%)	17 (30.4%)	–	–	5 (38.5)	5 (19.2)	–	–
Cause of liver disease	HBV	109 (85.8%)	29 (90.6%)	48 (85.8%)	χ^2^ = 4.116	0.533	10 (76.9%)	22 (84.6%)	χ^2^ = 5.813	0.214
	HCV	4 (3.2%)	0 (0.0%)	3 (5.4%)	–	–	0 (0.0%)	1 (3.8%)	–	–
	Alcohol	5 (3.9%)	1 (3.1%)	2 (3.6%)	–	–	0 (0.0%)	0 (0.0%)	–	–
	NAFLD	1 (0.8%)	0 (0.0%)	1 (1.8%)	–	–	1 (7.7%)	1 (3.8%)	–	–
	SC	3 (2.4%)	1 (3.1%)	0 (0.0%)	–	–	2 (15.4%)	0 (0.0%)	–	–
	None or other	5 (3.9%)	1 (3.1%)	2 (3.6%)	–	–	0 (0.0%)	2 (7.7%)	–	–

HBV, hepatitis B virus; HCV, hepatitis C virus; NAFLD, non-alcoholic fatty liver disease; SC, Schistosomal cirrhosis.

### 3.2 LI-RADS features

**Table 2** summarizes the consistency in the LI-RADS features assessed among different radiologists; excluding enhancing capsule (kappa value: -0.04~0.50, Kendall’s W value: 0.54) and iron sparing in solid mass (kappa value: -0.31~0.39, Kendall’s W value: 0.56), the consistency of the other features ranged from good to excellent, including non-peripheral washout (kappa value: 0.76~0.89, Kendall’s W value: 0.89), mosaic architecture (kappa value: 0.61-0.89, Kendall’s W value: 0.91), blood products in mass (kappa value: 0.90-0.95, Kendall’s W value: 0.95), and nodule-in-nodule architecture (kappa value: 0.82-0.91, Kendall’s W value: 0.92).

**Table 2:** LI-RADS features consistency of assessment among different radiologists.

LI-RADS features	Reader1 (%)	Reader2 (%)	Reader3 (%)	Reader1 vs. Reader2 *k* value (95% CI)	Reader1 vs. Reader3 *k* value (95% CI)	Reader2 vs. Reader3 *k* value (95% CI)	Kendall’s W
Nonrim APHE	110 (86.6%)	97 (76.4%)	112 (88.2%)	0.64 (0.35, 0.65)	0.82 (0.39, 0.76)	0.71 (0.15, 0.54)	0.81
Nonperipheral washout	80 (63.0%)	73 (57.5%)	72 (56.7%)	0.89 (0.80, 0.97)	0.87 (0.78, 0.96)	0.76 (0.65, 0.87)	0.89
Enhancing capsule	68 (53.5%)	60 (47.2%)	64 (50.4%)	0.50 (0.35, 0.65)	0.46 (0.31, 0.61)	-0.04 (-0.21, 0.13)	0.54
Threshold growth	2 (1.6%)	2 (2.4%)	2 (2.4%)	1	1	1	1
Mosaic architecture	55 (43.3%)	56 (44.1%)	65 (51.2%)	0.89 (0.81, 0.97)	0.84 (0.75, 0.94)	0.61 (0.77, 0.95)	0.91
Non-enhancing capsule	7 (5.5%)	7 (5.5%)	7 (5.5%)	0.70 (0.42, 0.98)	0.70 (0.42, 0.98)	0.70 (0.42, 0.98)	0.80
Blood products in mass	50 (39.4%)	48 (37.8%)	47 (37.0%)	0.90 (0.81, 0.97)	0.95 (0.90, 1.00)	0.92 (0.84, 0.99)	0.95
Fat in mass, more than adjacent liver	39 (30.7%)	42 (33.1%)	43 (33.9%)	0.84 (0.74, 0.94)	0.82 (0.71, 0.93)	0.73 (0.61, 0.86)	0.87
Nodule-in-nodule architecture	42 (33.1%)	43 (33.9%)	41 (32.3%)	0.91 (0.81, 0.97)	0.91 (0.83, 0.99)	0.82 (0.72, 0.93)	0.92
Corona enhancement	22 (17.3%)	29 (22.8%)	29 (22.8%)	0.83 (0.71, 0.95)	0.83 (0.71, 0.95)	0.78 (0.65, 0.91)	0.88
Restricted diffusion	110 (86.6%)	113 (89.0%)	108 (85.0%)	0.82 (0.66, 0.97)	0.76 (0.59, 0.93)	0.80 (0.66, 0.95)	0.86
Mild-moderate T2 hyperintensity	124 (97.6%)	120 (94.5%)	120 (94.5%)	0.59 (0.66, 0.97)	0.59 (0.66, 0.97)	0.40 (0.05, 0.73)	0.68
Fat sparing in solid mass	8 (6.3%)	15 (11.8%)	16 (12.6%)	0.67 (0.44, 0.89)	0.64 (0.41, 0.86)	0.45 (0.21, 0.68)	0.72
Iron sparing in solid mass	25 (17.9%)	20 (15.7%)	21 (16.5%)	0.33 (0.12, 0.53)	0.31 (0.11, 0.52)	0.39 (0.18, 0.60)	0.56
Transitional phase hypointensity	118 (92.9%)	117 (92.1%)	118 (92.9%)	0.72 (0.48, 0.95)	0.76 (0.54, 0.99)	0.72 (0.48, 0.95)	0.82
Hepatobiliary phase hypointensity	118 (92.9%)	117 (92.1%)	117 (92.1%)	0.72 (0.48, 0.95)	0.72 (0.48, 0.95)	0.67 (0.43, 0.92)	0.80
Subthreshold growth	11 (8.7%)	10 (7.9%)	10 (7.9%)	0.84 (0.67, 1.01)	0.89 (0.74, 1.04)	0.84 (0.67, 1.01)	0.91

Among the LI-RADS features, taking 2 cm as the cutoff value, tumors greater than 2 cm in size were found in 49 cases and 16 cases in the GPC-3-positive and -negative groups, respectively (P<0.001). In the GPC-3-positive and -negative groups, there was non-peripheral washout in 40 cases and 16 cases (P=0.044), enhancing capsule in 35 cases and 12 cases (P=0.024), mosaic architecture in 40 cases and 6 cases (P < 0.001), blood products in mass in 30 cases and 4 cases (P < 0.001), nodule-in-nodule architecture in 26 cases and 2 cases (P < 0.001), and restricted diffusion in 54 cases and 23 cases (P=0.003), respectively (**Table 3**).

**Table 3:** The univariate analysis between GPC-3 expression with MRI features and clinical factors in training cohort (categorical and dichotomized variables).

Variables	Characteristic	GPC-3(+)	GPC-3(-)	Test values	P values
Tumor size(cm)	<2.0cm	7 (12.5%)	16 (50.0%)	χ^2^ = 14.933	<0.001
	≥2.0cm	49 (87.5%)	16 (50.0%)	–	–
Cirrhosis	Absence	9 (16.1%)	8 (25.0%)	χ^2^ = 1.042	0.401
	Presence	47 (83.9%)	24 (75.0%)	–	–
Nonrim APHE	Absence	6 (10.7%)	6 (18.8%)	χ^2^ = 0.538	0.463
	Presence	50 (89.3%)	26 (81.3%)	–	–
Nonperipheral washout	Absence	16 (28.6%)	16 (50.0%)	χ^2^ = 4.041	0.044
	Presence	40 (71.4%)	16 (50.0%)	–	–
Enhancing capsule	Absence	21 (37.5%)	20 (62.5%)	χ^2^ = 5.115	0.024
	Presence	35 (62.5%)	12 (37.5%)	–	–
Threshold growth	Absence	55 (98.2%)	31 (96.9%)	–	1 (Fisher’s exact test)
	Presence	1 (1.8%)	1 (3.1%)	–	–
Mosaic architecture	Absence	16 (28.6%)	26 (81.3%)	χ^2^ = 22.651	<0.001
	Presence	40 (71.4%)	6 (18.8%)	–	–
Non-enhancing capsule	Absence	53 (94.6%)	30 (93.8%)	–	1 (Fisher’s exact test)
	Presence	3 (5.4%)	2 (6.3%)	–	–
Blood products in mass	Absence	26 (46.4%)	28 (87.5%)	χ^2^ = 14.489	<0.001
	Presence	30 (53.6%)	4 (12.5%)	–	–
Fat in mass, more than adjacent liver	Absence	39 (69.6%)	23 (71.9%)	χ^2^ = 0.049	0.825
	Presence	17 (30.4%)	9 (28.1%)	–	–
Nodule-in-nodule architecture	Absence	30 (54.6%)	30 (93.8%)	χ^2^ = 15.153	<0.001
	Presence	26 (46.4%)	2 (6.3%)	–	–
Corona enhancement	Absence	45 (80.4%)	26 (81.3%)	χ^2^ = 0.010	0.919
	Presence	11 (10.8%)	6 (18.8%)	–	–
Restricted diffusion	Absence	2 (3.6%)	9 (28.1%)	χ^2^ = 9.092	0.003
	Presence	54 (96.4%)	23 (71.9%)	–	–
Mild-moderate T2 hyperintensity	Absence	0 (0.0%)	2 (6.3%)	–	0.130 (Fisher’s exact test)
	Presence	56 (100.0%)	30 (93.8%)	–	–
Fat sparing in solid mass	Absence	53 (94.6%)	30 (93.8%)	–	1 (Fisher’s exact test)
	Presence	3 (5.4%)	2 (6.3%)	–	–
Iron sparing in solid mass	Absence	53 (94.6%)	28 (87.5%)	–	0.251 (Fisher’s exact test)
	Presence	3 (5.4%)	4 (12.5%)	–	–
Transitional phase hypointensity	Absence	2 (3.6%)	2 (6.3%)	–	0.620 (Fisher’s exact test)
	Presence	54 (96.4%)	30 (93.8%)	–	–
Hepatobiliary phase hypointensity	Absence	2 (3.6%)	4 (12.5%)	–	0.185 (Fisher’s exact test)
	Presence	54 (96.4%)	28 (87.5%)	–	–
Subthreshold growth	Absence	52 (92.9%)	30 (93.8%)	–	1 (Fisher’s exact test)
	Presence	4 (7.1%)	2 (6.3%)	–	–
TBIL (≤20.5μmol/L vs.>20.5μmol/L)	Absence	38 (67.9%)	23 (71.9%)	χ^2^ = 0.155	0.694
	Presence	18 (32.1%)	9 (28.1%)	–	–
DBil (≤6.8 μmol/L vs. >6.8 μmol/L)	Absence	27 (48.2%)	14 (43.8%)	χ^2^ = 0.163	0.686
	Presence	29 (51.8%)	18 (56.3%)	–	–
AST (38≤U/L vs.>38U/L)	Absence	33 (58.9%)	20 (62.5%)	χ^2^ = 0.108	0.742
	Presence	23 (41.1%)	12 (37.5%)	–	–
ALT (≤43U/L vs.>43U/L)	Absence	39 (69.6%)	24 (75.0%)	χ^2^ 0.287	0.592
	Presence	17 (30.4%)	8 (25.0%)	–	–
TP(≤60g/L vs.>60g/L)	Absence	9 (16.1%)	8 (250%)	χ^2^ = 1.042	0.307
	Presence	47 (83.9%)	24 (75.0%)	–	–
GLB(≤25g/L vs.>25g/L)	Absence	13 (23.2%)	7 (21.9%)	χ^2^ = 0.021	0.885
	Presence	43 (76.8%)	25 (78.1%)	–	–
PT(≤14s vs.>14s)	Absence	32 (57.1%)	19 (59.4%)	χ^2^ = 0.042	0.838
	Presence	24 (42.9%)	13 (40.6%)	–	–
PLT(≤125×10^9/L vs.>125×10^9/L)	Absence	24 (42.9%)	12 (37.5%)	χ^2^ = 0.242	0.623
	Presence	32 (57.1%)	20 (62.5%)	–	–
INR(≤1.15 vs.>1.15)	Absence	45 (80.4%)	25 (78.1%)	χ^2^ = 0.062	0.803
	Presence	11 (19.6%)	7 (21.9%)	–	–
AFP (>7 ng/ml vs. ≤7 ng/ml)	Absence	22 (39.3%)	13 (40.6%)	χ^2^ = 0.015	0.902
	Presence	34 (60.57%)	19 (59.4%)	–	–
CA242 (>20 iu/ml vs. ≤20 iu/ml)	Absence	54 (96.4%)	31 (96.9%)	–	1 (Fisher’s exact test)
	Presence	2 (3.6%)	1 (3.1%)	–	–
Child-Pugh score	A	39 (69.6%)	25 (78.1%)	Z=-0.930	0.353
	B	15 (26.8%)	7 (21.9%)	–	–
	C	2 (3.6%)	0 (0%)	–	–

TBIL, total bilirubin; DBil, Direct Bilirubin; AST, Aspartate aminotransferase; ALT, Alanine aminotransferase; TP, Total Protein; GLB, globulin; PT, Prothrombin time; PLT, platelet; INR, International normalized ratio; AFP, Alpha fetoprotein; CA242, Carbohydrate antigen 242.

### 3.3 Clinical indicators

There was no significant difference between the GPC-3-positive and -negative expression in the laboratory indicators of liver function, including TB, DBil, AST, ALT, TP and GLB (P>0.05); laboratory indicators of coagulation, including PT, PLT and INR (P>0.05); and laboratory indicators of tumor markers, including AFP, CA242, CYFRA211 and TSGF (P>0.05). The Fer levels in the GPC-3 positive and -negative groups were 344.39 µg/L ± 195.37 µg/L and 173.86 µg/L ± 135.42 µg/L, respectively, and the difference was statistically significant (P< 0.001) (**Tables 3**, **4**).

**Table 4:** The univariate analysis between GPC-3 expression with enhanced-MRI quantitative parameters and clinical factors in training cohort (continuous variables).

Variables	All cases	GPC-3(+)	GPC-3(-)	Test values	P value
PP-LNR	0.83 ± 0.21	0.81 ± 0.21	0.90 ± 0.24	t=1.849	0.006
PP-LMR	0.99 ± 0.23	1.02 ± 0.26	1.02 ± 0.18	t=0.022	0.983
LAP-LNR	1.26 ± 0.45	1.25 ± 0.41	1.25 ± 0.42	t=0.017	0.986
LAP-LMR	1.75 ± 0.64	1.75 ± 0.60	1.80 ± 0.71	t=0.335	0.738
PVP-LNR	0.99 ± 0.33	0.89 ± 0.29	0.98 ± 0.27	t=1.324	0.189
PVP-LMR	1.72 ± 0.61	1.72 ± 0.67	1.79 ± 0.61	t=0.496	0.621
TP-LNR	0.80 ± 0.21	0.75 ± 0.19	0.86 ± 0.21	t=-2.566	0.012
TP-LMR	1.49 ± 0.45	1.51 ± 0.45	1.53 ± 0.52	t=0.187	0.852
HBP-LNR	0.59 ± 0.23	0.53 ± 0.20	0.67 ± 0.24	t=2.784	0.007
HBP-LMR	1.39 ± 0.45	1.42 ± 0.44	1.42 ± 0.50	t=0.006	0.995
CER	0.59 ± 0.47	0.73 ± 0.42	0.40 ± 0.30	t=3.936	<0.001
CYFRA211	4.72 ± 3.05	4.78 ± 3.26	4.57 ± 2.68	t=0.303	0.763
TSGF	59.56 ± 16.99	60.39 ± 15.47	60.06 ± 17.68	t=0.090	0.929
Fer	300.88 ± 197.74	344.39 ± 195.37	173.86 ± 135.42	t=4.369	<0.001

PP, Pre-enhanced Phase; LAP, Late Artery Phase; PVP, Portal Venous Phase; TP, Transition Phase; HBP, Hepatobiliary Phase; LNR, Lesion-to-Normal parenchyma-Ratio; LMR, Lesion-to-Muscle-Ratio; CER, Contrast Enhancement Ratio; CYFRA211, cytokeratin fragment antiogen21-1; TSGF, Tumor-specific Growth Factor; Fer, ferritin.

### 3.4 Quantitative parameters of MRI enhancement

Among the quantitative parameters of MRI enhancement in the GPC-3 positive and -negative expression, PP-LNR was 0.81 ± 0.21 and 0.90 ± 0.24 (P=0.006), and TP-LNR was 0.75 ± 0.19 and 0.86 ± 0.21 (P= 0.012), respectively. The HBP-LNR was 0.53 ± 0.20 and 0.67 ± 0.24 (P=0.007), respectively, and the CER was 0.73 ± 0.42 and 0.40 ± 0.30 (P<0.001), respectively. The differences in PP-LNR, TP-LNR, HBP-LNR, and CER between the GPC-3-positive and -negative groups were statistically significant **Table 4**.

### 3.5 Multivariate logistic regression results of GPC-3 expression, establishment and validation of the nomogram model

**Figure 2:**
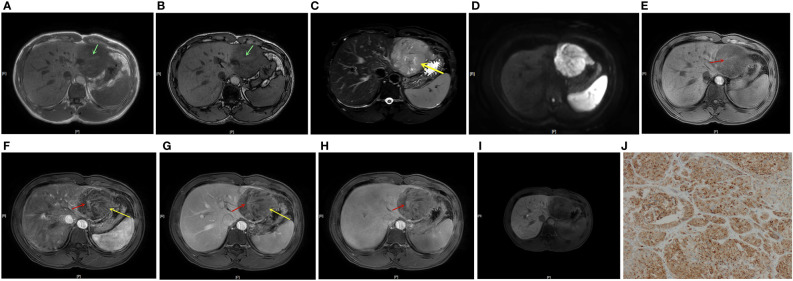
A 41-year-old male patient with HCC and positive GPC-3 expression. In-/out-of-phase T1WI showed a 7.1 cm-diameter mass in the left lateral lobe of the liver, with a strip-like hyperintensity in the hypointense background, indicating positive blood products in mass (**A, B**, green arrow). T2WI showed mild-moderate T2 hyperintensity in the lesion **(C)**. DWI showed restricted diffusion in the lesion **(D)**. Dynamic enhanced MRI showed positive nodule-in-nodule architecture in the lesion (**E-H**, red arrow, CER = 0.005). T2WI and the LAP and PVP images showed a mosaic architecture (**C, F, G** yellow arrow), and the TP and HBP scans showed a hypointense mass **(H, I)**. TP-LNR was 0.86, Fer was 178 ug/L, and the patient’s total nomogram points was 169 points, indicating that the preoperative risk for positive GPC-3 expression was 91%. The postoperative immunohistochemistry results showed positive GPC-3 expression **(J)**.

The factors with a p value < 0.1 in univariate results were included in multivariate logistic regression, using the stepwise backward method, and the results were as follows: blood products in mass, nodule-in-nodule architecture, mosaic architecture, CER, low TP-LNR, and Fer were independent predictors of positive GPC-3 expression, and the odds ratios (ORs) were 5.437, 10.682, 5.477, 11.788, 0.028, and 1.005, respectively **Table 5**; **Figure 2**.

**Table 5:** The multivariable logistic regression analysis for GPC-3 expression.

Variables	OR value	95% CI	β	SE	P value	χ^2^
Mosaic architecture	5.477	1.292-23.224	1.701	0.737	0.021	5.324
Blood products in mass	5.437	1.095-26.990	1.693	0.818	0.038	4.290
Nodule-in-nodule architecture	10.682	1.515-75.344	2.369	0.997	0.017	5.648
CER	11.788	1.244-111.709	2.467	1.147	0.032	4.623
TP-LNR	0.028	0.001-0.666	-3.584	1.621	0.027	4.887
Fer	1.005	1.000-1.009	0.005	0.002	0.037	4.333

CER, Contrast Enhancement Ratio; TP, Transition Phase; LNR, Lesion-to-Normal parenchyma-Ratio; Fer, Ferritin;OR, Odds ratio;CI, Confidence interval; SE, Standard error.

**Figure 3:**
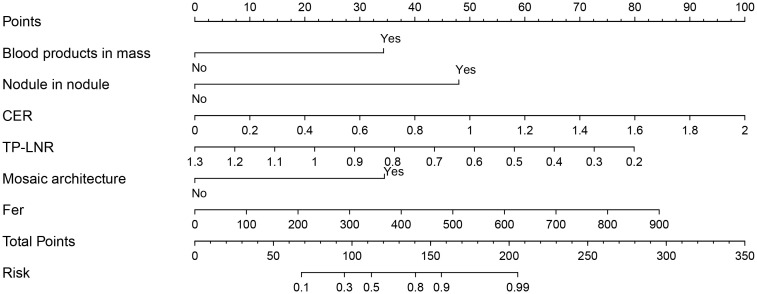
Nomogram constructed based on LI-RADS features, quantitative contrast-enhanced MRI parameters and clinical indicators for predicting GPC-3 expression. According to whether each patient had the features of blood products in mass, nodule-in-nodule architecture and mosaic architecture as well as the CER, TP-LNR, and Fer levels, for a total of 6 indicators, vertical lines can be drawn between each indicator and the nomogram points to obtain the value of each indicator. Then, the total score can be obtained by adding the scores of the 6 indicators; finally, a vertical line can be drawn between the total score and the nomogram total risk to predict the probability of positive GPC-3 expression in patients.

**Figure 4:**
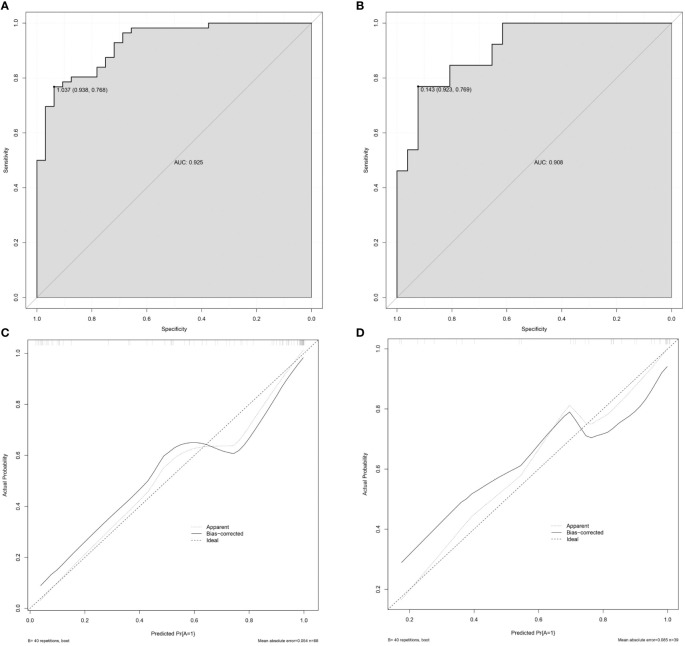
Validation of the nomogram. **(A)** The AUC of the prediction model in the training cohort was 0.925, 95% CI: 0.872–0.978. **(B)** The AUC of the prediction model in the validation cohort was 0.908, 95% CI: 0.816–1.000. **(C)** The calibration curve of the nomogram in the training cohort. **(D)** The calibration curve of the nomogram in the validation cohort. The x-axis is the predicted probability of the nomogram, and the y-axis is the actual probability of GPC-3 positive expression. The calibration prediction curve fits well with the standard curve, suggesting that the predicted probability of GPC-3 positive expression by the nomogram model is in good agreement with the actual probability. Note: ideal is the standard curve, apparent is the prediction curve, and bias-corrected is the calibration prediction curve.

The nomogram model for predicting the expression of GPC-3 in HCC was successfully established by R software (**Figure 3**). The total score ranged from 0-350 points and corresponded to the probability of positive GPC-3 expression in HCC patients, which ranged from 0.1-0.99. The area under the ROC curve of the nomogram model was 0.925 (95% CI, 0.872- 0.978) and 0.908 (95% CI, 0.816- 1.000) for the training cohort and validation cohort, respectively (**Figures 4A, B**). The calibration curve and the standard curve fit well (**Figures 4C, D**)The optimal threshold for predicting positive GPC-3 expression, according the Youden index, was 1.037, which had a sensitivity of 76.8% and specificity of 93.8% in the training cohort. In the validation cohort, these measures were 76.9% and 92.3%, respectively suggesting that the nomogram model based on LI-RADS features, quantitative MRI enhancement parameters and clinical indicators had good predictive performance.

## 4 Discussion

The immunotherapy approaches targeting GPC-3 include GPC-3 vaccines, anti-GPC-3 immunotoxins, combined therapy with immune checkpoint blockades (ICBs), and chimeric antigen receptor (CAR) T or NK cells. Existing clinical trials have confirmed that the abovementioned therapies have good application prospects (23, 24). Our study illustrates that blood products in mass, nodule-in-nodule architecture, mosaic architecture, CER, low TP-LNR, and Fer can be used as independent predictors of positive GPC-3 expression. The established nomogram can more accurately predict the expression of GPC3 and then hopefully noninvasively identify patients who are suitable for targeted GPC-3 immunotherapy.

In our study, the consistency between different radiologists in assessing LI-RADS features varied widely (kappa values ranged from -0.04 to 1, Kendall’s W values ranged from 0.54 to 1). However, the LI-RADS features (blood products in mass, nodule-in-nodule architecture, and mosaic architecture) that could independently predict GPC-3 positive expression had high consistency (kappa values ranged from 0.61 to 0.95, and Kendall’s W values ranged from 0.91 to 0.95). This is similar to the interreader agreement in previous studies on LI-RADS features (25–27). The mosaic architecture has been reported to be uncommon in nonhepatocellular carcinoma and is more common in HCC with a diameter greater than 3 cm (28). Some scholars believe that the mosaic architecture reflects the heterogeneity of tumors, corresponding to gross pathological hemorrhage, necrosis, cystic degeneration and tumor parenchyma, suggesting that the internal components of the lesions are complicated. Mosaic architecture is often seen in advanced HCC and can appear at the same time as the nodule-in-nodule architecture. Internal nodular enhancement corresponds to active tumor tissue and is related to the patient’s microvascular invasion (MVI) status and poor prognosis (29). Neovascularization in HCC promotes tumorigenesis and the development of metastasis and makes HCC a highly vascularized tumor. The tumor neovascularization is highly permeable, which makes the tumor prone to bleeding (30). Previous studies have shown that GPC-3 plays a key role in the occurrence and progression of HCC. GPC-3 can significantly promote the proliferation and differentiation of HCC tumor cells and regulate tumor angiogenesis and the immune microenvironment (21, 31). The above factors may explain why blood products in mass, nodule-in-nodule architecture, and mosaic architecture are independent predictors of GPC-3 expression.

Among the quantitative contrast-enhanced MRI parameters, CER and low TP-LNR were independent predictors of positive GPC-3 expression. We calculated the CER as the relative enhancement ratio between the LAP and the PP. LI-RADS believes that the peak enhancement of most HCC lies in the LAP, and it is recommended to evaluate the APHE of HCC in this phase (3). The HCC blood supply mostly arises from the systemic circulation, and GPC-3 can significantly promote HCC tumor angiogenesis (21). We believe that the above two factors may lead to a higher CER in HCC with positive GPC-3 expression. The TP is the ratio between the PVP and HBP and can reflect both the washout of contrast agents in HCC and the early uptake of Gd-EOB-DTPA by hepatocytes (3). Gd-EOB-DTPA is mainly taken up by hepatocytes through transporters expressed on the hepatocyte membrane. Organic anion transporting polypeptide (OATP) is the main carrier of Gd-EOB-DTPA uptake and is then excreted through multidrug resistance-related protein (MRP) to the bile (32). TP-LNR reflects the ratio of the signal in the lesion to that in the adjacent liver parenchyma, and the contrast agent washout in HCC occurs almost entirely in the TP. GPC-3-positive HCC has poor differentiation and poor prognosis, with downregulated OATP expression (33). The above factors determine the low signal of lesions in the TP and the high signal of the liver background, which may explain why a low TP-LNR was an independent predictor of positive GPC-3 expression. The study by Chen Y et al. (20) showed that a low PVP signal is an independent predictor of positive GPC-3 expression. The possible reason for the different results between studies is the different evaluation methods, as we used a quantitative method for measurement.

Among clinical indicators, Fer can be used as an independent predictor of positive GPC-3 expression. Previous studies have confirmed that the liver iron content of fibrotic livers is higher than that of normal liver, and the iron content of GPC-3-positive HCC patients is higher than that of GPC-3-negative patients, which may be related to the overexpression of transferrin receptors on the surface of HCC that cause a massive deposition of iron in the liver (21, 34, 35). The study by Chen Y et al. (20) showed that iron sparing in solid mass was an independent predictor of GPC-3 expression, and LI-RADS defined iron sparing in solid mass as a solid mass in the background of an iron-overloaded liver or inner nodule has lower iron concentration than siderotic outer nodule (3); thus, we think the results of our study is consistent with their result. A possible reason why iron sparing in solid masses was not significantly different between groups in our study is the difference in the interpretation of this features by different radiologists. In our study, the kappa values and Kendall’s W value of iron sparing in solid mass were 0.31~0.39 and 0.56, respectively.

There are some limitations in our study. First, this study is a single-center study, and it is necessary to continue to expand the sample size and conduct multicenter external verification in the future. Second, our prediction model has high specificity (93.8%) and moderate sensitivity (76.8%), which may help to identify HCC patients who are not suitable for GPC-3-targeted immunotherapy in the future, but whether this model can be combined with other indicators to improve its sensitivity without impairing specificity in predicting GPC-3 expression warrants further research. Moreover, to ensure matching between the target lesions and pathological results, this study excluded patients with multiple tumors and only included patients with a single intrahepatic tumor. The application of this predictive model for the expression of GPC-3 in multiple intrahepatic lesions deserves further study. Finally, this study only developed a relatively convenient and feasible prediction model for GPC-3 expression but did not predict the prognosis of patients who receive CAR-T cells targeting GPC-3 as treatment for advanced HCC. As a new generation of immunotherapy for HCC, this approach deserves further in-depth study.

In conclusion, we developed a nomogram model constructed with LI-RADS features, quantitative contrast-enhanced MRI parameters, and clinical indicators that can accurately and conveniently and noninvasively predict the expression of GPC-3 in HCC patients. This model can help identify potential patients suitable for GPC-3-targeted immunotherapy, which is beneficial for individualized treatment and avoids unnecessary immunotherapy-related adverse reactions.

## Data availability statement

The original contributions presented in the study are included in the article/supplementary material, further inquiries can be directed to the corresponding author.

## Ethics statement

Written informed consent was not obtained from the individual(s) for the publication of any potentially identifiable images or data included in this article.

## Author contributions

Conception and design: YS, J-kS, and X-wZ; administrative support: J-kS; provision of study materials or patients: QY, RZ, and WH; collection and assembly of data: YS, Y-yZ, X-jQ, and ZZ; data analysis and interpretation: Y-yZ, C-gW, and QY; manuscript writing: YS. All authors contributed to the article and approved the submitted version.
